# Effects of Thermal Treatment on Natural Clinoptilolite-Rich Zeolite Behavior in Simulated Biological Fluids

**DOI:** 10.3390/molecules25112570

**Published:** 2020-05-31

**Authors:** Oana Cadar, Marin Senila, Maria-Alexandra Hoaghia, Daniela Scurtu, Ion Miu, Erika Andrea Levei

**Affiliations:** 1 National Institute for Research and Development of Optoelectronics Bucharest INOE 2000, Research Institute for Analytical Instrumentation, 67 Donath Street, 400293 Cluj-Napoca, Romania; oana.cadar@icia.ro (O.C.); marin.senila@icia.ro (M.S.); alexandra.hoaghia@icia.ro (M.-A.H.); daniela.scurtu@icia.ro (D.S.); 2 SC UTCHIM SRL, 12 Buda Street, 240127 Ramnicu Valcea, Romania; utchim_vl@yahoo.com

**Keywords:** natural zeolite, clinoptilolite, thermal treatment, simulated gastric fluid

## Abstract

This study presents the effect of thermal treatment (450, 500, 600, 750, and 800 °C) on a Romanian clinoptilolite-rich natural zeolite, along with the interaction of raw and thermally treated zeolites with simulated gastric fluid (SGF, pH = 1.20) at different zeolite to SGF ratios and exposure times. The zeolites were characterized using gravimetric analysis, X-ray fluorescence, powder X-ray diffraction (pXRD), and Fourier transform infrared (FT-IR) spectroscopy. The chemical composition of the zeolite subjected to thermal treatment did not change significantly with the increase of temperature. Structural changes were not detectable by pXRD and FT-IR analyses in the zeolites thermally treated up to 500 °C, while above 600 °C a gradual structural breakdown of zeolite was noticed. At high temperatures, the broad, low-intensity peaks in pXRD patterns indicated the partial amorphization of the crystalline structure. The pXRD and FT-IR analyses showed that the crystalline structure of zeolites remains unaffected after their exposure to SGF. The results revealed that the amounts of Fe, Na, Mg, K, Ca, Al, and Si released depends mainly on the zeolite to SGF ratio, and to a lower extent on the thermal treatment temperature, while the exposure time of 1 to 7 days does not have a significant impact on the elements released in SGF.

## 1. Introduction

Zeolites are crystalline aluminosilicates with three-dimensional open frameworks built by SiO_4_ and AlO_4_ tetrahedra linked by oxygen bridges and orderly distributed intracrystalline cavities, molecular sizes micropores, or pore channels. Generally, the framework structure (Si:Al ratio) together with the crystal shape and size determine the physical and chemical properties of zeolites [1]. The Structure Commission of the International Zeolite Association (IZA) categorizes zeolites based on the Si:Al ratio into low silica zeolites (Si:Al molar ratio = 1–2; SiO_2_:Al_2_O_3_ mass ratio = 1.18–2.35), medium silica zeolites (Si:Al molar ratio = 3–10; SiO_2_:Al_2_O_3_ mass ratio = 3.53–11.76), and high silica zeolites (Si:Al molar ratio > 10; SiO_2_:Al_2_O_3_ mass ratio > 11.76). Zeolites with a low Si:Al molar ratio are hydrophilic, while zeolites with a high Si:Al ratio are hydrophobic [1,2,3].

Worldwide, about 70 natural zeolite types have been identified and more than 260 zeolites have been synthesized. The largest producers of natural zeolites are China, South Korea, Jordan, Turkey, and Japan. In Romania, the volcanic tuff deposits from the Neogene age from intramountain basins (Transylvania, Sighet, Silvania, Strei, and Getic) contain 15 natural zeolite species, with clinoptilolite, heulandite, stilbite, laumonite, natrolite, and mesolite being more frequently found [4,5]. However, the zeolite exploitation in Romania is low.

The natural zeolites are used mainly as dietary supplements, ion exchangers, and in environment remediation, while the synthetic zeolites are used mainly as detergent builders, catalysts, and absorbents or desiccants [6,7]. Recently, zeolites have attracted significant interest due to their ion exchange capacity, crystallinity, thermal stability, and well-defined cage structure, making them good candidates for a wide range of promising applications in the fields of water purification, fuel cells, renewable energy production, and storage [8,9,10].

The chemical and thermal treatments are the most used techniques to modify the zeolite’s characteristics by controlling their structural and morphological properties [1,4,8]. These treatments allow the removal of impurities; the enhancement of sorption properties, surface area, and porosity; and also determine important crystallinity loss [8]. The majority of zeolites can be dehydrated without a major change in the crystal structure, followed by their rehydration through water adsorption from the atmosphere or proximate liquid phase. However, full dehydration may cause irreversible structural changes or structural collapse by weakening the linkages in the framework structure. Furthermore, through dehydration, some exchangeable cations located in the channels could become trapped [11,12]. With thermal treatment, the zeolite structure may undergo the following changes: (i) cell-volume reduction through the elimination of water or template organic molecules (dehydration and calcination processes); (ii) reconstructive or displacive phase transformations; (iii) breakage and formation of T-O-T bonds; (iv) negative thermal expansion; (v) partial structural breakdown; and (vi) structural collapse (amorphization or recrystallization). The thermal behavior of zeolites is controlled by the Si:Al ratio; framework topology; ionic potential; size of exchangeable cations; and the number, nature, and coordination of extra-framework cations [11,13].

There are a high number of studies that report the properties of natural zeolites from different deposits after alkaline (NaOH, KOH), acidic (HCl, H_3_PO_4_, H_3_BO_3_), and thermal treatments [8,14,15,16,17]. Additionally, the beneficial properties of purified zeolites in human and veterinary medicine were recently reported [4,18]. Natural zeolites (mainly clinoptilolite) were successfully used for organism detoxification, as antidiarrheal and antiacid agents, and as adjuvant in anticancer therapy. The addition of zeolites to feeds enhanced the immunity of new-born ruminants and the gastrointestinal absorption of toxic compounds, reduces the deposition of radioactive compounds and the oxidative stress, boosting the growth performance of animals [17,19]. Due to their ability to encapsulate different ions and molecules, zeolites may be used in drug formulation as carriers for oral drugs and gene delivery. They may also be used for biosensing, hemostasis, tissue engineering, implant coating, and wound healing [18,19,20]. Moreover, zeolites have significant antioxidant, hepatoprotective, and anti-inflammatory effects. Beside the favorable effects of natural zeolites, some of them may present cytotoxicity or carcinogenicity, or may disturb the mineral metabolism favoring the retention of some elements, such as Al or Si [18]. Despite all the beneficial and adverse effects that have been determined for zeolites, data on their biologic effects is still scarce. The use of natural zeolites in nutrition and medicine depends on their performance under digestive conditions. The few studies on the behavior of natural zeolites in physiological systems are insufficient to reflect the complex digestion media [19,21,22]. In this regard, this paper aims to study the influence of thermal treatment at various temperatures on the structure of a clinoptilolite-type natural zeolite and the release of Si, Al, Fe, Ca, Mg, K, Na, and P from zeolite in simulated gastric fluid (SGF, pH = 1.20).

## 2. Results

### 2.1. Chemical Composition of the Zeolites

The obtained results (Na_2_O, K_2_O, CaO, SiO_2_, Al_2_O_3_, Fe_2_O_3_, loss on ignition (LOI)) for the certified reference material (CRM) are in good agreement with the certified values (Table 1).

The slight increase of major metal oxides (Na_2_O, K_2_O, CaO, MgO, SiO_2_, and Al_2_O_3_) in thermally treated zeolite (TZ) samples is attributed to the removal of water molecules from the zeolite structure. The increasing trend is not so obvious for oxides found in low amounts (Fe_2_O_3_, MnO, and TiO_2_). LOI progressively decreases with increasing thermal treatment temperature because the clinoptilolite gradually loses its rehydration ability, the zeolite structure being progressively affected [23,24]. The slight variations in the Si:Al ratio probably depend only on the method uncertainty of the chemical analyses. Similar results were reported by Duvarci et al. for a natural clinoptilolite zeolite from Turkey that was thermally treated between 700 and 1200 °C [25]. Furthermore, de Gennaro et al. reported that a high Fe_2_O_3_ content results in color darkening [24]. In our case, the color change from light beige to light brown with increasing thermal treatment temperature confirm the presence of Fe_2_O_3_ and may influence the selection of further applications in the field.

### 2.2. Structure of the Zeolites

**Figure 1:**
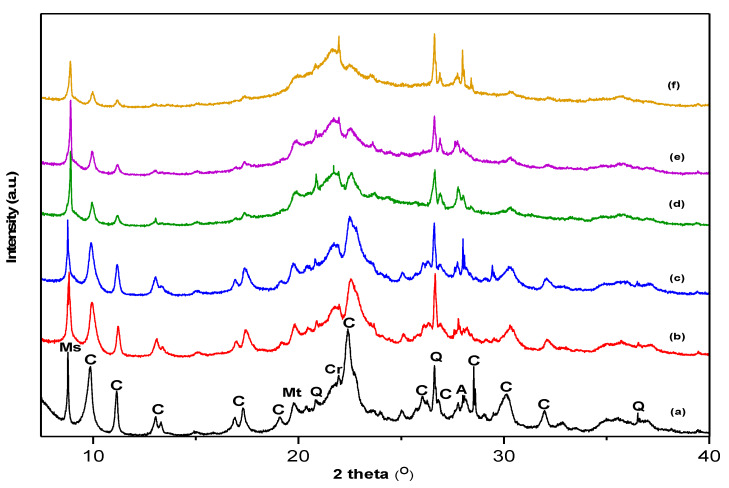
The powder X-ray diffraction (pXRD) patterns of (a) raw zeolite (RZ), (b) zeolite thermally treated at 450 °C, (c) zeolite thermally treated at 500 °C, (d) zeolite thermally treated at 600 °C, (e) zeolite thermally treated at 750 °C, and (f) zeolite thermally treated at 800 °C. Note: clinoptilolite, C; muscovite, Ms; quartz, Q; montmorillonite, Mt; cristobalite, Cr; albite, A.

Natural zeolites never exist in a unique form and usually consist of several minerals. The pXRD patterns of the zeolite samples show the characteristic peaks of clinoptilolite at 2θ values of 9.86, 11.16, 22.46, 26.03, 29.99, and 31.95° (Figure 1). According to pXRD analysis, the initial raw zeolite (RZ) contains clinoptilolite (PDF 01-089-7539) as the major crystalline phase, accompanied by muscovite (PDF 00-034-0175), quartz (SiO_2_) (PDF 00-005-0490), montmorillonite (PDF 00-058-2038), SiO_2_ (α-cristobalite, tetragonal structure, PDF 00-039-1425), and albite (PDF 00-020-0548). The non-crystalline components were not quantified by the pXRD analysis, but the presence of amorphous volcanic glass in RZ (Figure 1a) is indicated by the broad diffraction hump in the region 2θ = 18–25°.

Generally, the thermal treatment results in a loss in crystallinity. In our study, the relative degree of crystallinity progressively decreases by increasing the thermal treatment temperature from 65% (TZ-450) to 24% (TZ-800), indicating the gradual breakdown of the structure. Similar crystallinity losses by thermal treatment were reported for natural zeolites from Cuba and Armenia [26,27]. The thermal treatment of RZ up to 500 °C does not produce significant structural changes detectable by pXRD, while a further increase in the temperature produces a gradual collapse of the zeolite. The structure damage is indicated by the decrease of clinoptilolite peaks, which are more visible for the main peak at 22.46°. At 600 °C (TZ-600), the partial collapse of the clinoptilolite structure is indicated by the decrease of the characteristic peaks and the formation of SiO_2_ (β-cristobalite, cubic structure, PDF 00-027-0605), as indicated by the appearance of the peak at 21.71°. At 800 °C (TZ-800), the sharp peak at 27.99° attributed to anorthite (PDF 01-070-0287) with the calcium silicate structure indicates the further collapse of the zeolite structure and the formation of a new phase. The total disappearance of the characteristic peaks of clinoptilolite indicating its complete amorphization did not take place up to 800 °C. The slightly change of the other peaks’ height could be related to the variation in the composition. The increase of thermal treatment temperature up to 800 °C leads to an increasing amount of amorphous phase as shown by the amorphous hump at 2θ = 20–25°. The thermal behavior of zeolites depends on the complex interactions of the framework with the cations and water molecules from the channel network [13]. The thermal stability up to 500 °C of the natural clinoptilolite-rich zeolite from Chilioara, Romania, can be explained by the reversible dehydration that takes place with little or no modification of the crystal structure [13]. However, the preliminary structural modifications induced by thermal treatments are driven by the loss of water molecules, which occurs concomitantly with the migration of extra-framework cations in order to achieve the optimal bonding after water molecules leave the structure [13]. The breakdown of the zeolitic structure at high temperatures was also reported by Duvarci et al. for a zeolitic tuff containing mainly clinoptilolite quarried from Turkey, which was thermally treated in the range of 200–1200 °C [24]. On the other hand, in both RZ and TZ samples, the intensity and position of the peak attributed to crystalline quartz (SiO_2_) at 26.62° did not change with the increase of temperature. In contrast, the partial breakdown of the structure at 600 °C for a natural clinoptilolite originating from Cuba was reported by Arcoya et al., and the collapse at 550 °C of zeolites belonging to earth alkali clinoptilolite with low alkali content by Akkoka et al. [8,26].

In all cases, the pXRD analysis showed that the crystalline structure of zeolites remains unaffected after their exposure to SGF. Additionally, no other phases were noticed and no loss in the crystallinity of zeolites occurred after the immersion in SGF. However, a significant increase of the intensity of peaks characteristic to quartz for samples exposed to SGF was observed. A possible explanation could be the purification effect of HCl by solubilization of mineral impurities from the quartz crystal lattice structure [26,28]. Arcoya et al. reported a decrease of the characteristic peaks by HCl treatment, however our results show no modification of the characteristic peak intensities, probably due to the stability of crystal structure [26,29]. Our results are in line with those of Kavak et al., who reported that the characteristic peak intensities of clinoptilolite in the control sample were nearly the same as those of samples exposed to gastric fluids [19]. 

**Figure 2:**
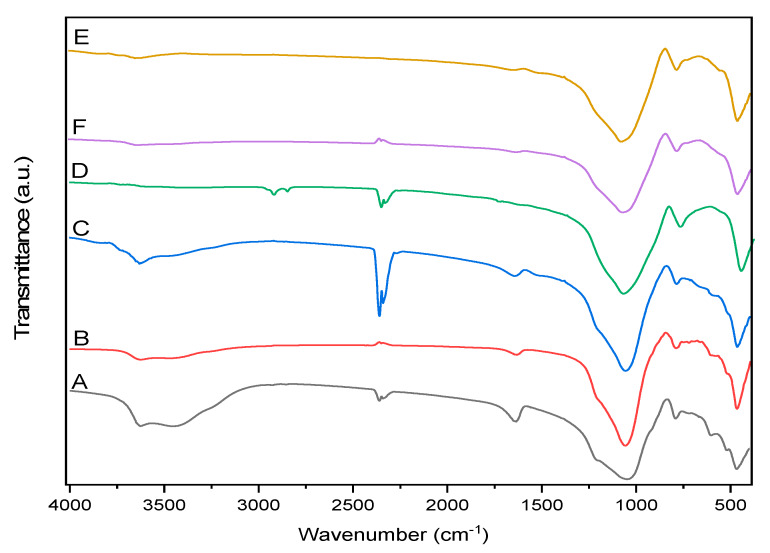
Fourier transform infrared (FT-IR) spectra of (A) raw zeolite (RZ), (B) zeolite thermally treated at 450 °C, (C) zeolite thermally treated at 500 °C, (D) zeolite thermally treated at 600 °C, (E) zeolite thermally treated at 750 °C, and (F) zeolite thermally treated at 800 °C.

The most intense peak in the FT-IR spectra of the RZ and TZ-450-TZ-800 (Figure 2) appears at 1050 cm^−1^ and is characteristic to clinoptilolite, being assigned to T-O bond (T = Si and Al) vibration. This peak shifts to higher wavenumbers (1090 cm^−1^) with the increase of the thermal treatment temperature as a result of the dehydration process [2,24,30,31,32]. Other characteristic peaks of the clinoptilolite appear at 786 and 606 cm^−1^ and are assigned to Si-O-Si and O-T-O bond vibration, respectively. The intensity of the vibration band from 606 cm^−1^ is proportional to the amount of clinoptilolite present in the zeolites. The decrease of this peak in TZ-600 indicates that the clinoptilolite structure starts to collapse at 600 °C, while the presence of this peak in the TZ-800 sample suggests that the collapse is not complete at 800 °C. Duvarci et al. [25] also reported that the structure of clinoptilolite collapse at 800 °C.

The band at 672 cm^−1^ is attributed to the vibrations in the TO_4_ tetrahedron, while the low-intensity band at 720 cm^−1^ is attributed to symmetric vibrations of Si-O-Al bonds [32]. The intensity of this bands decreases with increasing temperature, confirming the dehydration process. The bands at 524 cm^−1^ correspond to the vibrations of pore opening, at 467cm^−1^ to internal vibrations of TO_4_ tetrahedron, while at 1640 cm^−1^ to Si-O bond vibration; these bands also disappear at temperatures above 600 °C [30]. The bands in the range of 1600–3700 cm^−1^ are attributed to the presence of water in the zeolite structure [31]. The peak of variable intensity that appears around 2250–2500 cm^−1^ in all cases is attributed to CO_2_ adsorbed by the samples during the cooling, after thermal treatment. The band at 3420 cm^−1^ is attributed to the vibration of H bonds between water molecules and oxygen atoms at the network surface, to the vibration of Si-OH groups fixed through hydrogen bonds in the network defects, and to the vibration of loosely bonded water molecules from the network surface [2,31]. The presence of functional -OH groups is indicated by the band at 3622 cm^−1^ [30]. The intensity of these bands decreases and then disappears at high temperatures due to dehydration. No changes in the zeolites structure were indicated by the FT-IR spectra of samples exposed to SGF.

### 2.3. pH Changes

In all cases, only a low increase of pH values after the immersion of zeolites in SGF (pH = 1.20, up to 1%) was observed. However, no relationship could be established even after long exposure times, with the small changes being in the range of the pH determination method’s uncertainty.

### 2.4. Concentration of Major Elements in Simulated Gastric Fluid after Immersion of Zeolites

**Figure 3:**
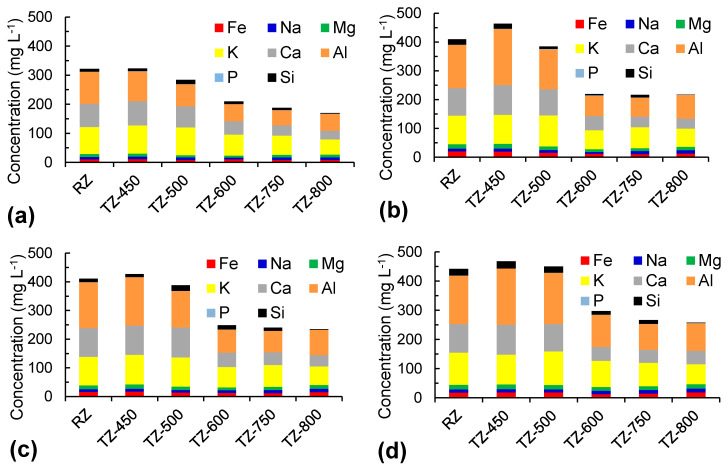
Influence of the thermal treatment temperature on element dissolution with a 10:1 (*w*:*v*) zeolite:simulated gastric fluid (SGF) ratio for exposure times of (**a**) 1 day, (**b**) 3 days, (**c**) 5 days, and (**d**) 7 days.

The interactions between zeolites and SGF could be attributed to several physicochemical reactions, including ion exchange, hydrolysis, dissolution, potential surface precipitation, and sorption [20,32]. In acidic media such as SGF, considering the high affinity of the zeolites toward H^+^, the proton is adsorbed onto the zeolites’ surfaces, determining the release of other cations [32,33]. Figure 3 presents the elements release in SGF from zeolites treated at different temperatures after different exposure times. For all exposure times, the total elements released from zeolites treated at low temperatures are higher than from zeolites treated at high temperatures. No differences in the metal releasing patterns of RZ and samples thermally treated at temperatures up to 500 °C (RZ, TZ-450, and TZ-500) were observed. Additionally, the metal release of samples thermally treated at temperatures above 600 °C (TZ-600, TZ-750, TZ-800) was comparable. However, between the samples treated at low and high temperatures, significant differences were found, with the metal release being much lower in samples thermally treated at high temperatures. This difference in the metal release could be attributed to the partial breakdown of the clinoptilolite above 500 °C, together with the intense dehydration, which can lead to cell volume reduction and to exchangeable cations trapping in the zeolite channels [13].

For all thermal treatment temperatures, Al, Ca, and K were the elements released in the highest concentrations. As known, the Ca and K release takes place via the exchange of Ca and K with H^+^ [8]. The exchange capacity of K is higher than that of Ca in acidic media, as the ionic potential increases in the order of K < Na < Ca < Mg [8]. The decrease of the Si, Ca, and Al release in SGF with increasing temperature above 600 °C could be explained by the progressive collapse of the zeolite structure and the start of the anorthite formation that contains these elements. Except for 1-day exposure time, where a lower metal release was observed, for the other exposure times no important differences were observed. In all cases, the Al release was at least 10 times the Si release, indicating that dealumination is a more intense process than desilication in acidic media. According to the theory of Brønsted and Lewis, the dissolution process of natural zeolites is influenced by the presence of H^+^ and OH^−^ ions in the solution, resulting from their acidic and basic behaviors. However, the interactions of natural high-silica zeolites (such as clinoptilolite) with acidic aqueous media generally occur at low dissolution rates, as they are acid resistant [34]. The Al release from zeolites under acidic conditions was also investigated by Akkoca et al. for Turkish natural zeolites [8] and by Selvam et al. in Cuban natural zeolites [35]. The high Al release is determined by the easier hydrolysis of Al-O-Al than of Si-O-Si bonds under acidic conditions and the formation of complexes on the zeolite surface in the presence of Cl^−^ [8].

**Figure 4:**
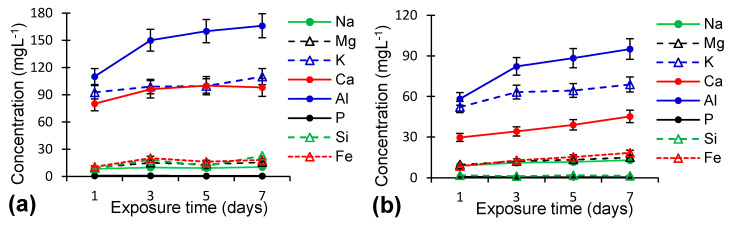
Influence of exposure time on the element dissolution with a 10:1 (*w*/*v*) zeolite/SGF ratio for (**a**) raw zeolite (RZ), (**b**) zeolite thermally treated at 800 °C.

Except for Al, the exposure time (Figure 4) had a low influence on the element release for any of the studied zeolites, as the aggressive attack of highly acidic solutions such as SGF weakened the zeolite’s structure, favoring the rapid (hours) release of weakly linked cations from the zeolite pores and their replacement with protons [33,36]. This release determined the framework instability and subsequent release of Al and then of Si from the aluminosilicates, as Al is more susceptible to acidic attack than Si [33]. The dissociated Si and Al, together with the other solubilized elements, determined the formation of an amorphous aluminosilicate layer on the zeolite surface, which protected the structure from further attacks [33]. In our samples, after the first day of exposure, equilibrium between dissolution and readsoption had already occurred and no further important changes took place.

**Figure 5:**
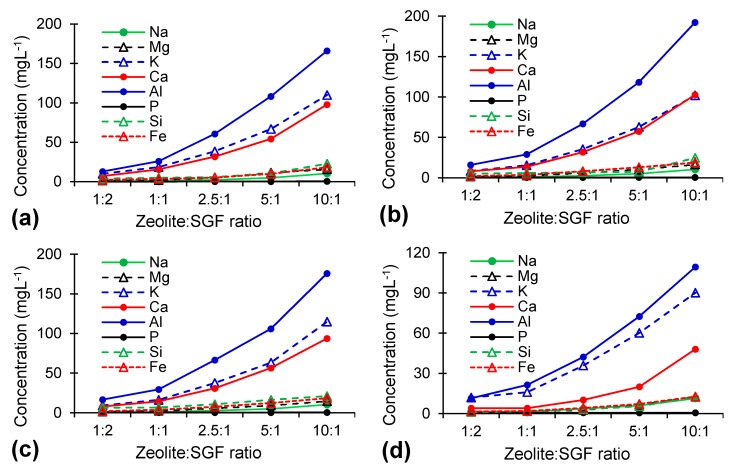

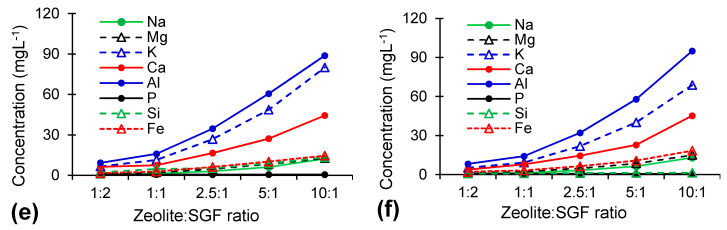
Influence of zeolite:SGF (*w*:*v*) ratio on the element dissolution with an exposure time of 7 days for (**a**) raw zeolite (RZ), (**b**) zeolite thermally treated at 450 °C, (**c**) zeolite thermally treated at 500 °C, (**d**) zeolite thermally treated at 600 °C, (**e**) zeolite thermally treated at 750 °C, and (**f**) zeolite thermally treated at 800 °C.

The increase of the elements released with the increasing zeolite to SGF ratio (Figure 5) was observed for all studied elements except P, as well as for all thermal treatments. This increase is sharper for Al, K, and Ca than for the other elements, as these elements are released preferentially from the zeolite structure.

**Figure 6:**
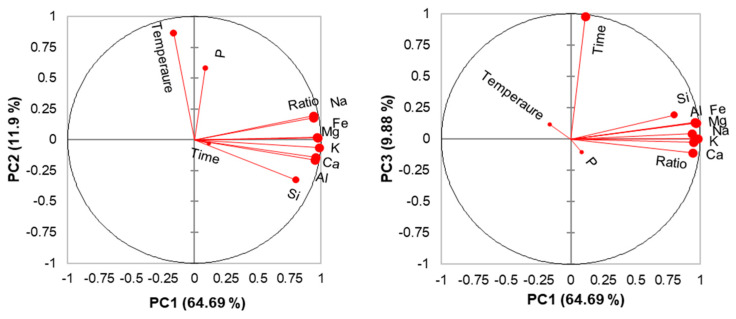
Principal component analysis (PCA) correlation charts.

The principal component analysis (PCA) on standardized data of elements solubilized in SGF showed 11 principal components (PCs), of which the first 3 PCs have eigenvalues above 1 and explain about 86% of the data variability. The 3 PCs are associated with the zeolite to SGF ratio (PC1, 64.7%), thermal treatment temperature (PC2, 11.9%), and exposure time (PC3, 9.9%). The relationships between the variables and PCs are shown in the orthogonal representation of the PCs (Figure 6). 

As shown by the representation of PC1 and PC2, the concentrations of Fe, Na, Mg, K, Ca, Al, and Si increase with increasing zeolite to SGF ratio, while only Si, Ca, Al, and K slightly decrease and P increases with increasing thermal treatment temperature, probably due to the structural changes caused by the thermal treatment [13]. The other elements are not influenced by the temperature used for the thermal treatment. Usually, Si and Al do not undergo ion exchange, but under the influence of strong acids zeolite desilication and dealumination may occur [37]. The relationship between PC1 and PC3 shows that the P concentration is not influenced by the zeolite to SGF ratio or by the exposure time. However, the concentrations of the other elements are slightly influenced by the exposure time, probably because in acidic solutions such as SGF (pH = 1.2), the released Na, K, Ca, and Mg ions from zeolites rapidly (in the first 30 min) reach an equilibrium after 5 h [35]. Additionally, the release of Si and Al ions occurs within the first 30 min and gradually increases up to 5 h, mainly due to the desilication and dealumination of the zeolite [35]. Thus, the exposure time of 1 to 7 days does not significantly change the cation release.

## 3. Materials and Methods

### 3.1. Thermal Treatment

The clinoptilolite-type zeolite was collected from the Chilioara quarry, located in Northern Romania [9]. The tuff was crushed and sieved to obtain a particle size smaller than 100 µm, washed with distilled water to remove the soluble impurities, and dried in an oven at 105 °C (RZ). The RZ was further thermally treated at 450 °C (TZ-450), 500 °C (TZ-500), 600 °C (TZ-600), 750 °C (TZ-750), and 800 °C (TZ-800) for 4 h in air. As the decomposition of organic compounds still occurs at 450 °C, it was selected as starting thermal treatment temperature.

### 3.2. Characterization

The powder X-ray diffraction (pXRD) patterns were recorded at room temperature using a D8 Advance (Bruker, Karlsruhe, Germany) diffractometer operating at 40 kV and 40 mA with CuK_α_ radiation (λ = 1.54060 Å). The data were collected in the 2θ range of 10–40°, with a step size of 0.01° and a counting time of 1 s per step. The degree of crystallinity was estimated from the relative intensities of the most characteristic peaks of clinoptilolite, taking as reference the intensity of these reflections in the RZ sample [26]. Fourier transform infrared (FT-IR) spectra were collected using a Spectrum BX II (Perkin Elmer, Waltham, MA, USA) spectrometer on 1% KBr pellets measuring 12 mm in diameter, in the range of 4000–400 cm^−1^ using a resolution of 2 cm^−1^. The contents of Al, Fe, Ca, Mg, K, Na, Mn, and Ti in RZ and TZ samples were determined using a S1 Titan 800 X-ray fluorescence spectrometer (Bruker, Berlin, Germany). The conversion to the corresponding oxide was made by multiplying the element concentration with 1.8895 (Al_2_O_3_), 1.4297 (Fe_2_O_3_), 1.3392 (CaO), 1.6583 (MgO), 1.2046 (K_2_O), 1.3480 (Na_2_O), 1.2912 (MnO), and 1.6683 (TiO_2_) [29]. The SiO_2_ and loss of ignition (LOI) were determined by gravimetry [38]. A potash feldspar (BCS-CRM 376/1) certified reference material (Bureau of Analyzed Samples, Middlesbrough, UK) with a similar matrix to zeolite samples was analyzed for quality control purposes.

### 3.3. Behaviour of the Zeolites in Simulated Gastric Fluid

As the simulated gastric fluid (SGF, pH = 1.2), an aqueous solution of 0.1 M HCl without enzymes was used [21]. The deionized water used for all experiments was obtained from a Milli-Q Plus water purification system (Millipore, Bedford, MA, USA). Zeolite samples were exposed to SGF solution under mild shaking conditions at 37 ± 0.5 °C in 1:2, 1:1, 2.5:1, 5:1, and 10:1 zeolite-to-SGF ratios (*w*:*v*) for 1, 3, 5, and 7 days. After immersion, the liquid phase was separated by centrifugation at 4000 rpm for 5 min and filtered through a 45 µm cellulose acetate membrane. The concentrations of Si, Al, Fe, Ca, Mg, K, Na, and P released in SGF were measured using a 5300 Optima DV (Perkin Elmer, Waltham, MA, USA) inductively coupled plasma optical emission spectrometer (ICP-OES) after microwave digestion using the method described previously [9]. For the ICP-OES, external calibration using calibration solutions in the range of 0–20 mg L^−1^ were prepared from 1000 mg L^−1^ multielement (Na, K, Ca, Mg, Fe and Al) and monoelement (P and Si) standard solutions (Merck, Darmstadt, Germany) diluted in 0.5% (*v*:*v*) HNO_3_ [39]. The experiments were carried out in triplicate and the average values were reported. The pH changes of SGF before and after exposure of zeolites were monitored using a Seven Excellence multiparameter (Mettler Toledo, Schwerzenbach, Switzerland) [9].

### 3.4. Statistical Analysis

In order to reveal the main parameters that influence the concentrations of elements released from zeolite exposed to SGF, principal component analysis (PCA) with varimax rotation of the principal components (PCs) with eigenvalues higher than 1 was applied, using the XLSTAT (Addinsoft, Paris, France) Microsoft Excel add-on software (BASIC+, 2019.3.2).

## 4. Conclusions

The effect of thermal treatment on natural Romanian clinoptilolite-rich zeolite and its behavior in simulated gastric fluid (SGF, pH = 1.20) were investigated. The chemical compositions of zeolites did not change significantly, but an important loss in crystallinity was observed with increasing thermal treatment temperature. Both pXRD and FT-IR data indicated that the structure collapse and the transformation of clinoptilolite into an amorphous phase starts at 600 °C, progressing gradually at higher temperatures, but is not completed until 800 °C. The overall results showed that the structural stability of zeolites remained unaffected after their exposure to SGF. The metal releasing pattern was similar for untreated and thermally treated zeolites up to 500 °C, but for zeolites heated above 600 °C the metal release had a notable decrease. The main elements released were K, Al, and Ca. The main factor that influenced the metal release in SGF was the zeolite to SGF ratio, with the thermal treatment influencing the Si, Ca, Al, K, and P release only to a low extent. The exposure time had little effect on metal release, except for Al. Due to the fact that the original crystalline structure of RZ remained unchanged up to 500 °C, the zeolitic tuff can be used for applications that require moderate temperatures. Additionally, the color change by thermal treatment should be taken into consideration for the selection of the potential application fields.

## Figures and Tables

**Table 1:** Chemical compositions (wt.%) of the raw zeolite (RZ) and zeolites thermally treated at 450 °C (TZ-450), 500 °C (TZ-500), 600 °C (TZ-600), 750 °C (TZ-750) and 800 °C (TZ-800) together with the certified (CRM-c) and experimental (CRM-e) results obtained for potash feldspar (BCS-CRM 376/1) certified reference material.

Zeolite	Na_2_O	K_2_O	CaO	MgO	SiO_2_	Al_2_O_3_	Fe_2_O_3_	MnO	TiO_2_	Si:Al	LOI
RZ	0.92	3.12	1.66	0.87	72.95	12.79	1.05	0.03	0.18	7.90	6.42
TZ-450	1.09	3.33	1.71	0.83	73.40	12.84	0.96	0.04	0.17	7.92	5.57
TZ-500	1.08	3.63	1.81	0.93	72.91	12.95	1.06	0.03	0.18	7.80	5.39
TZ-600	1.06	3.76	1.96	1.02	72.80	13.52	1.04	0.04	0.17	7.46	4.58
TZ-750	1.21	3.68	2.09	1.15	73.40	13.50	1.09	0.03	0.16	7.53	3.66
TZ-800	1.25	3.76	1.98	1.23	73.62	14.06	1.10	0.04	0.19	7.25	2.72
CRM-c	3.00	11.59	0.421	-	65.77	18.63	0.085	-	-	-	-
CRM-e	2.96	11.51	0.413	-	65.63	18.70	0.079	-	-	-	-
